# Verbascoside: Identification, Quantification, and Potential Sensitization of Colorectal Cancer Cells to 5-FU by Targeting PI3K/AKT Pathway

**DOI:** 10.1038/s41598-018-35083-2

**Published:** 2018-11-16

**Authors:** Yasmeen M. Attia, Dina M. El-Kersh, Hebatallah A. Wagdy, Mohamed M. Elmazar

**Affiliations:** 1 Pharmacology Department, Faculty of Pharmacy, The British University in Egypt, El-Sherouk City, P.O. Box 43, Cairo, 11837 Egypt; 2 Pharmacognosy Department, Faculty of Pharmacy, The British University in Egypt, El-Sherouk City, P.O. Box 43, Cairo, 11837 Egypt; 3 Pharmaceutical Chemistry Department, Faculty of Pharmacy, The British University in Egypt, El-Sherouk City, P.O. Box 43, Cairo, 11837 Egypt; 4 The Center for Drug Research and Development (CDRD), Faculty of Pharmacy, The British University in Egypt, El-Sherouk City, P.O. Box 43, Cairo, 11837 Egypt

**Keywords:** Cancer therapeutic resistance, Cancer therapeutic resistance

## Abstract

Colorectal cancer (CRC) is the third most common cancer mortality worldwide. Although, 5-Fluorouracil (5-FU)-based chemotherapeutic regimens remain the mainstay for treatment of CRC, intrinsic and acquired resistance to 5-FU is the main reason for treatment failure and relapse. Adjunct or add-on therapy, therefore, should be thought of to enhance responsiveness to 5-FU. Verbascoside (VER) is a phenylethanoid glycoside ingredient present in many *Plantago* species and was widely used in traditional medicine. VER showed antiproliferative effects in many cancer types including CRC. In the present study, VER in *Plantago* seeds was identified using UPLC-MS/MS and quantified using newly developed and validated UPLC-DAD followed by investigating its potential sensitization of CRC cells to 5-FU *in vitro*. The potential impact on PI3K/AKT pathway was also investigated. A synergistic cytotoxic interaction between 5-FU and VER besides G1 cell cycle arrest were detected. Enhanced apoptosis mainly by affecting Bax and Bcl-2 and to a lesser extent Bcl-xL and p53 was also observed. Additionally, 5-FU combined to VER was capable of significantly reducing PI3K and p-AKT/total AKT ratio. Overall, these results suggest a potential role of VER as an adjuvant treatment to decrease the resistance of CRC cells to 5-FU possibly by targeting the PI3K/AKT pathway.

## Introduction

Colorectal cancer (CRC) is the third most prevalent form of cancer and is a leading cause of cancer mortality worldwide^1^. Correlation between colitis and inflammatory bowel disease and development of CRC was established long time ago^2^. An estimated increased incidence of almost 1% each year was also suggested in previous studies^3^.

Host inflammatory and immune responses play a major role in the pathogenesis of the disease, given the microenvironment where dysplasia associated with colitis and CRC develop. The underlying mechanism through which CRC develops as a consequence of chronic inflammation is thought to be driven by a number of cytokines leading to enhanced proliferation of altered epithelial cells^4^.

The phosphoinositide 3-kinase (PI3K)/AKT signalling pathway has been implicated in the pathogenesis of several cancer types and hence drugs targeting this pathway were being extensively investigated in recent years^5^. PI3K/AKT signalling pathway enhances cell division and inhibits apoptosis. Physiologically, the activation of PI3K/AKT signalling pathway is tightly controlled and relies on the activation of specific growth signal receptors. However, hyperactivation of PI3K/AKT pathway caused by genetic alterations has been reported in many types of cancer including CRC. These genetic aberrations have been observed in almost 40% of colorectal tumors^5,6^.

Cancer chemotherapy remains the mainstay for treatment of CRC. Using 5-fluorouracil (5-FU) in treatment protocols had greatly enhanced survival rates in many cancer types, yet the largest impact was mainly observed with CRC^7^. 5-FU is known to exert its cytotoxic effect by interfering with the folate pathway consequently interfering with DNA and RNA synthesis in cancer cells^8^. However, the response of CRC to 5-FU in the advanced stage is only limited to a maximum of 15%. In spite the fact that survival rates were improved after adding oxaliplatin and irinotecan to treatment protocols, several toxicities associated with 5-FU were still reported^9^. Among these toxicities are myelosuppression, mucositis, diarrhea, and hand-foot syndrome^10^.

Genus *Plantago*, the major in family Plantaginaceae comprises almost 275 species. Different *Plantago* species were widely used since decades in traditional medicine. The most popular beneficial ingredient of *Plantago* genus is the polysaccharide mucilage present in the dorsal epidermal layer of the seeds known as *husk*^11^. A clinical study was conducted to determine the efficacy of using the *husk* obtained from *Plantago pysillium* L. in treatment of type II Diabetes. The results were favored for *Plantago pysillium* L. *husk* in lowering serum levels of glucose, triglycerides and cholesterol^12^. Beside the *husk*, the seeds of genus *Plantago* included other major phytochemical ingredients such as the phenylethanoids class, a cinnamic acid derivative. Interest in phenylethanoid glycosides has been growing recently due to their significant role in protecting against and treating a wide range of human disorders. Verbascoside (VER), also known as acteoside, is a phenylethanoid glycoside that was first isolated from mullein (*Verbascum sinuatum* L.; Scrophulariaceae)^13^ but is also found in several other plant species among which is the *Plantago pysillium* L.^14^. VER is hydrophilic in nature and has various pharmacological activities such as antioxidant, antimicrobial, anti-inflammatory, neuroprotective, and wound healing effects along with antiproliferative effects in cancer^15–17^. VER was previously recognized for its potent anti-proliferative, pro-apoptotic, and prodifferentiative chemopreventive / chemotherapeutic potential^18^. It showed anti-tumor activity *in vitro* in human gastric carcinoma by inducing cancer cell differentiation and apoptosis *via* telomerase–dependent modulation^19^. Similarly, it was also evident that VER could induce differentiation of leukemic cells towards the monocyte-macrophage lineage^18,19^.

In the present study, a targeted identification and quantification of the phenylethanoid glycoside, VER, present in both *Plantago pysillium* L. and *Plantago afra* L. were achieved by UPLC-MS/MS and UPLC-DAD. Efficacy of VER, alone and combined with 5-FU, against CRC was further assessed *in vitro*. Moreover, potential targeting of PI3K/AKT signalling pathway by the combination treatment was also investigated.

## Material and Methods

### Chemicals

Verbascoside (VER) reference standard was purchased from Carl Roth GmbH + Co. KG-Karlsruhe, Germany. Seeds of *Plantago pysillium* L. and *Plantago afra* L. were obtained from Haraz Company of herbal products, Giza, Egypt. Methanol (MeOH) (HPLC grade), Formic Acid (FA), glacial acetic acid (Ac A), and 5-FU were purchased from Sigma-Aldrich, Germany.

### Instrumentation

The UPLC system used was Thermo Fisher UHPLC Dionex Ultimate 3000 (Germering, Germany): the pump (ISO-3100SD), the autosampler (WPS 3000 SL), the column thermostat (TCC-3000 SD), Diode Array detector (DAD-3000RS) and the software utilized for data acquisition was Chromeleon 6.8 (Germering, Germany). The column utilized was Acclaim^TM^ RSLC 120 C18 (2.1 × 100 mm) with 2.2 μm particle size. The UPLC-ESI-MS/MS system consisted of ACQUITY UPLC H-Class system, Xevo^TM^ TQD triple-quadrupole tandem mass spectrometer with an electrospray ionization (ESI) interface (Waters Corporation, Milford, MA, USA). The column employed was Acquity BEH C18 (2.1 × 100 mm) with 1.7 μm particle size. Water purification system (Thermo scientific Barnstead Smart2Pure 3 UV, Hungary) was utilized to prepare deionized water. For solvent degasing Clifton Ultrasound bath (Clifton, London, United Kingdom) was employed.

### Plant extraction

Fifty grams of seeds of *P. pysillium* L. and *P. afra* L., each finely grinded and extracted with 80% hydromethanol. The hydromethanolic extract was evaporated at reduced pressure with rotary evaporator (BÜCHI, Germany) at 45 °C and kept in refrigerator till further analyses.

### Identification of verbascoside using UPLC-ESI-MS/MS

For identification of VER in *P. pysillium* L. and *P. afra* L. using UPLC-MS/MS system, the optimum conditions were as follows: The mobile phase consisted of the same two solvents; (A): Deionized water containing 0.1% formic acid and (B): Methanol containing 0.1% formic acid. The flow rate was 0.4 ml/min and the gradient time (t_G_) was 24 min: isocratic with 10% B for 0–0.6 min, after gradient from 10% B to 90% B from 0.6 to 20 min, afterwards gradient 90% B to 100% B from 21–22 min and lastly the initial conditions 10% B from 22–24 min. The parameters for analysis were carried out using negative ion mode as follows: source temperature 150 °C. Desolvation gas temperature 400 °C, cone voltage 30 eV, capillary voltage 3 kV. The flow rate of Nitrogen gas as cone gas and desolvation gas was 50 and 900 L/h, respectively. Mass spectra were detected in the ESI negative ion mode between m/z 100–900. The peaks and spectra were processed using the Maslynx 4.1 software and tentatively identified by comparing its retention time and mass spectrum with reported data.

### Identification and quantification of verbascoside using UPLC-DAD

For separation and quantification of VER in *P. pysillium* L. and *P. afra* L. using UPLC-DAD system, the optimum conditions were as follows: The mobile phase consisted of two solvents; (A) Deionized water containing 0.1% formic acid and (B) Methanol containing 0.1% formic acid. The gradient time (t_G_) was 20 min: Isocratic with 10% B for 0–0.6 min, after gradient from 10% B to 50% B from 0.6 to 6 min, then isocratic with 50% B from 6 to 11 min, afterwards gradient 50% B to 90% B from 11–13 min and kept for 2 minutes, finally back to the initial conditions 10% B from 15–17 min and remained at this condition for 3 min. The flow rate was 0.4 ml/min, the temperature was 35 °C, the injection volume was 10 μl and the UV detector was adjusted at 332 nm. It is worth mentioning that the mobile phase solvents; in both systems, were filtered using 0.22 μm filter and then degased for 15 min before utilization.

The identification of the VER; in both plant extracts; was carried out by comparing the retention time with its standard. While the quantification of VER was accomplished utilizing the established calibration curve.

### The standard and the plant extract preparation

One mg/ml stock of VER standard was prepared by dissolving 10 mg VER in 10 ml MeOH. Working solution (0.1 mg/ml) was prepared by dilution from the stock solution using MeOH. Ten mg of either *P. pysillium* L. or *P. afra* L. was dissolved in 10 ml methanol then ultrasonicated for 15 min and filtered with 0.22 μm syringe filter.

### Method validation

The method was validated according to the ICH guidelines^20^ and the following parameters were investigated: Linearity was evaluated by constructing a calibration curve; in which the area was plotted versus the concentration of the VER corresponding standards. In order to establish the calibration curve, 10 VER standards (6, 8, 10, 20, 40, 60, 80, 100, 200, and 400 μg/ml) were prepared by serial dilution from the stock solution. Each one of these standards was injected three times on the system. The limit of quantification is the concentration at which standard deviation of the intercept/slope ratio was equal to 10 and the limit of detection is the concentration at which standard deviation of the intercept/slope ratio was equal to 3.3. Repeatability of the method was assessed by injecting three concentrations (10, 20, and 40 μg/ml) for 3 times on the same day and then relative standard deviation (% RSD) was calculated. Meanwhile, intermediate precision was evaluated by injecting the same three concentrations for three consecutive days and the % RSD was determined. Accuracy was evaluated by comparing the actual found concentration by the system to the theoretical value of ten standards of the calibration curve. The results were expressed in terms of percentage recovery (%R) and % RSD. Further investigation for the accuracy took place by spiking 1 ml of 0.1 mg/ml of each plant extract with known concentration of standard (10, 20, and 30 μg/ml). Each sample; for each plant; was injected three times then the % recovery and the % RSD were calculated.

### Cell lines and cell culture

Colorectal cancer cell lines; Caco-2 and HCT-116 were obtained from the American Type Culture Collection (ATCC, VA, USA). Cells were cultured using Dulbecco’s Modified Eagle Medium (DMEM; Invitrogen, Life Technologies, CA, USA) supplemented with 10% fetal bovine serum (FBS; GE Healthcare HyClone, WV, USA), streptomycin (100 μg/ml) and penicillin (100 units/ml) in a humidified atmosphere of 5% CO_2_ at 37 °C. Cells were serially passaged at 80–90% confluency.

### Cytotoxicity assay

Caco-2 and HCT-116 cells were seeded in 96-well plates. Twenty-four hours later, cells were treated with 5-FU (0.01–100 µM), VER (0.01–100 µM), 5-FU (0.01–100 µM) + low dose of VER (0.01 µM), 5-FU (0.01–100 µM) + high dose of VER (0.1 µM), or Dimethylsulphoxide (DMSO; Sigma-Aldrich, St. Louis, MO, USA) as a vehicle. Cytotoxicity test was performed using 3-(4,5-dimethylthiazol-2-yl)−2,5-diphenyltetrazolium bromide (MTT) assay (Sigma-Aldrich, St. Louis, MO, USA) according to the manufacturer’s instructions, as previously described^21^. Briefly, cells were seeded in 96-well plates and cultured overnight. The following day, cells were treated with the above concentrations of 5-FU and/or VER as well as DMSO. Seventy-two hours later, 20 μl of MTT was added to each well followed by a 2 h incubation period after which the absorbance was measured. Cell viability was calculated as a percentage of the absorbance at 570 nm in treated cells against that of control. All experiments were performed in triplicates. Half-maximal inhibitory concentration (IC_50_) was calculated using GraphPad Prism software, version 5.00 (GraphPad Software, CA, USA). For studying the nature of drug interaction, the combination index (CI) was calculated according to the following formula^22^:

$${\rm{C}}{\rm{.I}}{\rm{.}}=[{{\rm{IC}}}_{50}{({\rm{A}})}_{{\rm{pair}}}{/\mathrm{IC}}_{50}({\rm{A}})]+[{{\rm{IC}}}_{50}{({\rm{B}})}_{{\rm{pair}}}{/\mathrm{IC}}_{50}({\rm{B}})]$$

The interaction was described as synergistic if CI < 1; antagonistic if CI > 1; and additive if CI equals to 1.

### Apoptosis assay using Annexin V/Propidium iodide staining

Caco-2 cells cultured in six-well plates were treated with 10 µM of 5-FU + 0.1 µM of VER for 48 hours. Then, the cells were fixed in cold ethanol for half an hour and stained with 5 μl Annexin V-FITC and 5 μl propidium iodide (PI) using an Annexin V-FITC Apoptosis Detection Kit (BioVision, CA, USA). The cells were then placed at room temperature for 15 min in the dark then analyzed by a FACScan flow cytometer (Beckman Coulter, CA, USA). Apoptosis was evaluated in terms of the FITC-positive cells.

### Cell cycle analysis

Forty-eight hrs following the exposure of Caco-2 cells to 10 µM of 5-FU + 0.1 µM of VER, cells were trypsinized and washed with PBS, resuspended in cold methanol, and kept overnight at 4 °C. Collected cells were then resuspended in sodium citrate buffer together with RNase, and incubated at 37 °C for 30 min. After centrifugation, cells were resuspended in PBS and filtered. Cell cycle analysis was then performed using FACSCalibur Flow Cytometer (BD Biosciences, San Jose, CA, USA).

### Gene and protein expression experimental design

Caco-2 cells were divided into three groups: (1) Blank control group where cells were treated with the vehicle; DMSO, (2) 5-FU group where cells were treated with 10 µM of 5-FU, (3) VER group where cells were treated with 0.1 µM of VER, and (4) 5-FU + VER group where cells were treated with 10 µM of 5-FU + 0.1 µM of VER. All treatments were started 24 hrs after cells were seeded in culture flasks. Gene expression was assessed at 48 hrs of treatment exposure, while protein expression was assessed at 72 hrs of exposure.

### RNA extraction and real time quantitative polymerase chain reaction (RT-qPCR) assay

Total RNA was isolated using RNeasy Mini Kit (Qiagen, USA). The iScript^TM^ One-Step RT-PCR Kit with SYBR^®^ Green (Bio-Rad, CA, USA) was used for cDNA synthesis and PCR amplification steps, according to the manufacturers’ instructions, as described previously^23^. The relative mRNA expression levels [fold change from untreated control samples normalized to glyceraldehyde phosphate dehydrogenase (GAPDH) as the housekeeping gene] of p53, Bcl-2 (B-cell lymphoma 2), Bax (Bcl-2 associated X protein), and Bcl-xL (B-cell lymphoma-extra large) were assessed using the 2^−ΔΔCt^ analysis method, as previously described by Livak and Schmittgen^24^. The primer sequences used for RT-qPCR are listed in Table 1.

**Table 1:** Sequence of primers used for quantitative real time polymerase chain reaction (qPCR) and their National Center for Biotechnology Information (NCBI) accession numbers.

Primer	Sequence	NCBI Accession number
**p53**	Forward: 5′-CCCCTCCTGGCCCCTGTCATCTTC-3′ Reverse: 5′-GCAGCGCCTCACAACCTCCGTCAT -3′	**NM_001276696.1**
**Bax**	Forward: 5′-GTTTCATCCAGGATCGAGCAG-3′ Reverse: 5′-CATCTTCTTCCAGATGGTGA-3′	**NM_001291429.1**
**Bcl-2**	Forward: 5′-CCTGTGGATGACTGAGTACC-3′ Reverse: 5′-GAGACAGCCAGGAGAAATCA-3′	**NM_000657.2**
**Bcl-xL**	Forward: 5′-GATCCCCATGGCAGCAGTAAAGCAAG-3′ Reverse: 5′-CCCCATCCCGGAAGAGTTCATTCACT-3′	**NM_001322240.1**
**GAPDH**	Forward: 5′-TGCCTCCTGCACCACCAACT-3′ Reverse: 5′-TGCCTGCTTCACCACCTTC-3′	**NM_001289746.1**

### Determination of caspase-3, -8, and -9 activity

In order to determine the potential of treatments to induce apoptosis and investigate the underlying mechanism of action, levels of active caspase-3, caspase-8, and caspase-9 were measured using human caspase-3, caspase-8, and caspase-9 ELISA kits obtained from Invitrogen (CA, USA), according to the manufacturers’ instructions.

### Determination of total PI3K activity

To determine the effect of 5-FU, VER, and 5-FU + VER on total PI3K activity, human PI3K ELISA kit (MyBioSource, CA, USA) was used according to the manufacturer’s instructions. Absorbance was measured at 450 nm.

### Determination of total AKT and phospho-AKT (Ser473)

To determine the effect of 5-FU, VER, and 5-FU + VER on total AKT (t-AKT), and phosphorylated AKT (p-AKT) activity; ELISA kit for the determination of t-AKT and p-AKT (Ser473) (Abcam, MA, USA) was used according to the manufacturer’s instructions. Briefly, cells were seeded in 96 well-culture plates and after 24 hrs, the cells were exposed to the different treatments. 72 hrs later, media was removed and both t-AKT and p-AKT were determined in cell lysates. The p-AKT/total AKT ratio was then calculated.

### Statistical analysis

All values are presented as means ± standard deviation (S.D.) from three independent experiments performed in triplicates. Statistical analysis was performed by one-way ANOVA followed by Bonferroni *post hoc* test using GraphPad Prism, version 5.0 (GraphPad Software, CA, USA) (v5). Statistical significance was determined at P < 0.05.

## Results and Discussion

### Identification and suggested fragmentation of verbascoside

**Figure 1:**
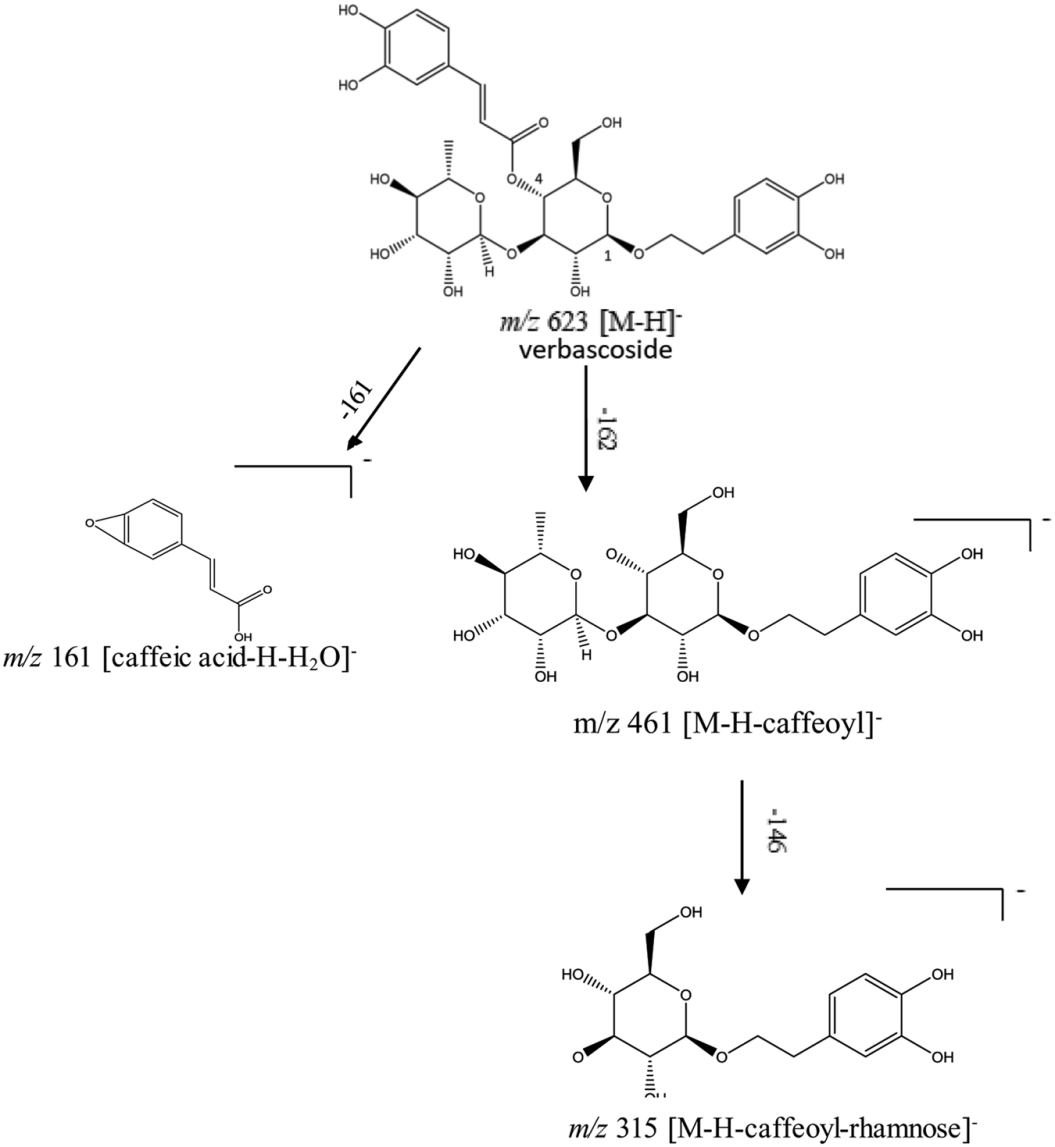
Verbascoside fragmentation pattern.

**Figure 2:**
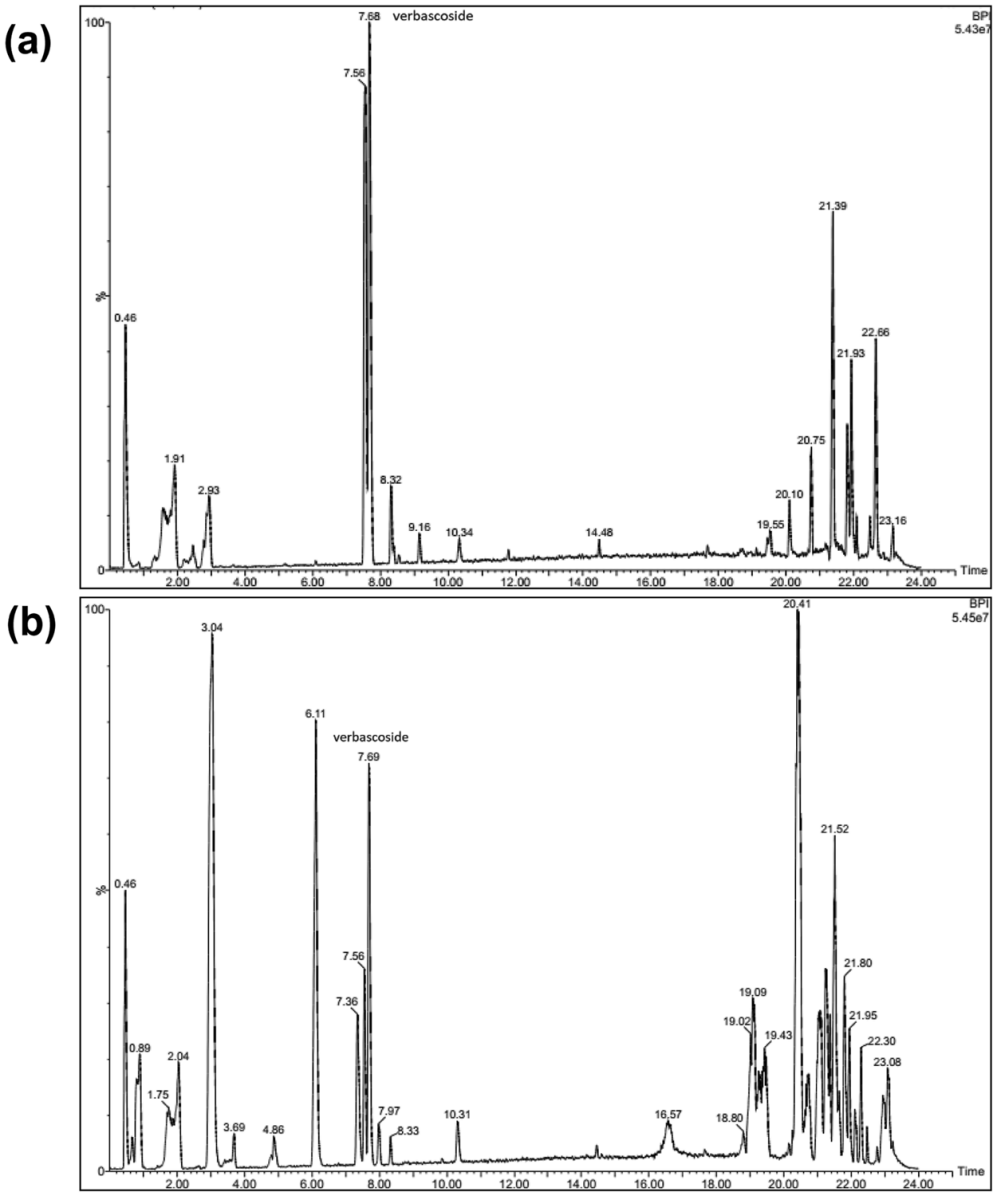
Total ion chromatogram (TIC) of total methanolic extract of seeds of (**a**) *P. pysillium* L. and (**b**) *P*. *afra* L.

**Figure 3:**
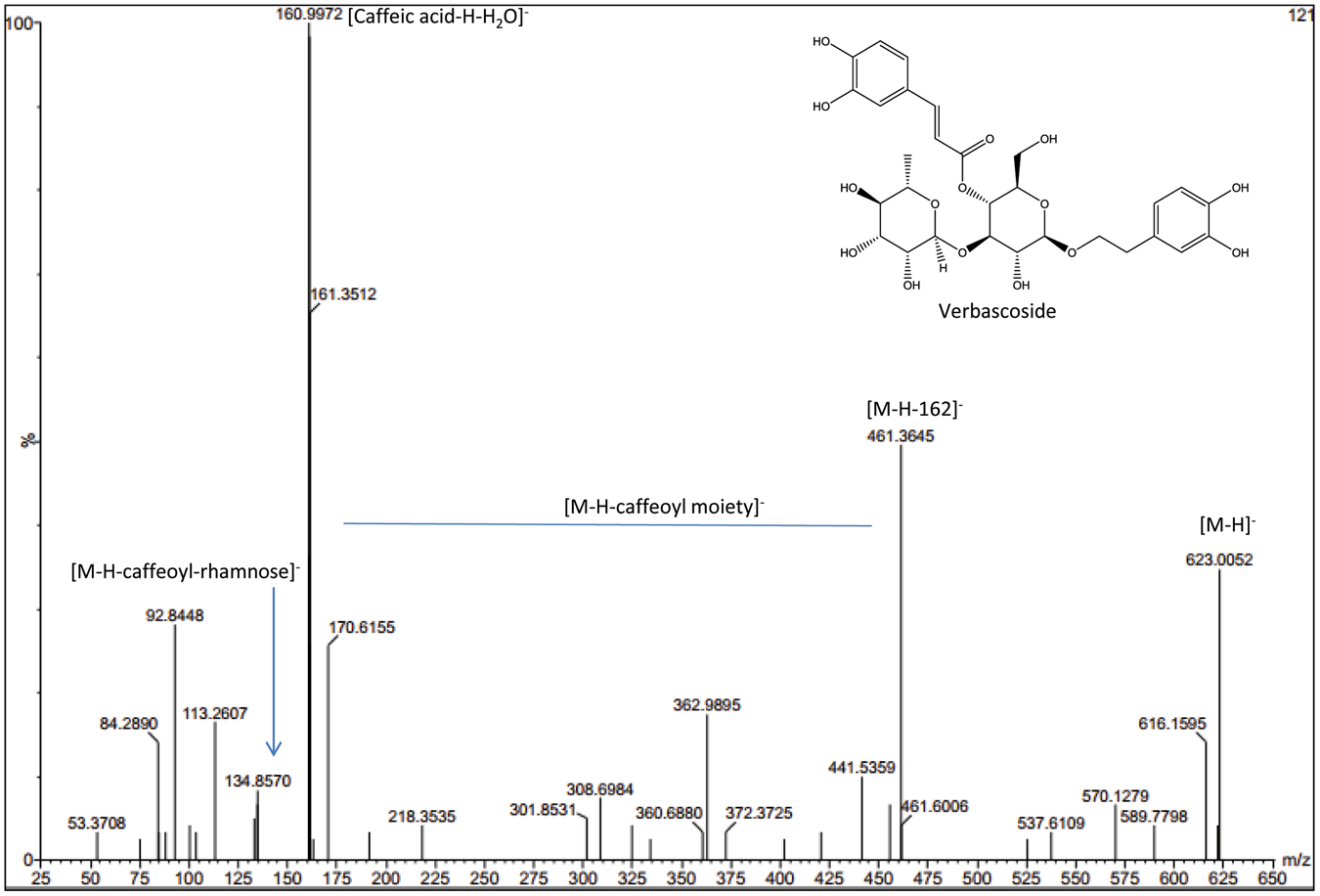
Daughter ion spectrum of verbascoside detected in seeds of *P. pysillium* L. and *P. afra* L.

Vebascoside (VER) or acteoside, a phenylethanoid glycoside composed mainly of a sugar skeleton of *β*-glucose and rhamnose monosaccharaides whereas the caffeoyl and hydroxyl phenylethyl aglycons replaced the hydroxyl groups of C_4_ and C_1_ of *β*-glucose, respectively as shown in the structure in Fig. 1. Total ionization chromatogram in the negative mode (TIC) of both *Plantago* species revealed the presence of VER at retention times 7.68 and 7.69 min. of *P. pysillium* L. and *P. afra*, L., respectively as in Fig. 2 a,b. Identification of VER through its fragmentation was observed in the presented fragmentation as shown in Fig. 1 and the daughter ion spectrum as shown in Fig. 3. The presence of molecular ion at *m/z* 623 (C_29_H_35_O_15_)^−^ for VER [M-H]^−^ structure whereas characteristic ion peak of the phenyl propanoid moiety recognized at *m/z* 161 [caffeic acid-H-H_2_O]^−^ was due to loss of H_2_O from caffeic acid at *m/z* 179 [C_9_H_7_O_4_]^−^ and the ion peak at *m/z* 461 represents [M-H-hexose sugar]^−^ due to loss of a hexose sugar (−162 amu)^25–27^. An ion peak at *m/z* 135 could be an evidence of anhydrophenethanol moiety^27^ as shown in Fig. 1.

### Method development and validation of VER on UPLC-DAD

Several methods have been investigated to reach the optimum method. At the beginning, different solvents were investigated; water 0.1% acetic acid and MeOH, then doubling the concentration of % acetic acid was also investigated. After, another solvent was tested; water 0.1% formic acid and MeOH and finally the best resolution and least retention time were achieved using water 0.1% formic acid and MeOH 0.1% formic acid. Accordingly, it was selected as the optimum solvent. It is worth mentioning that doubling the concentration of formic acid did not have a dramatic effect on the resolution or the retention time (tR).

**Figure 4:**
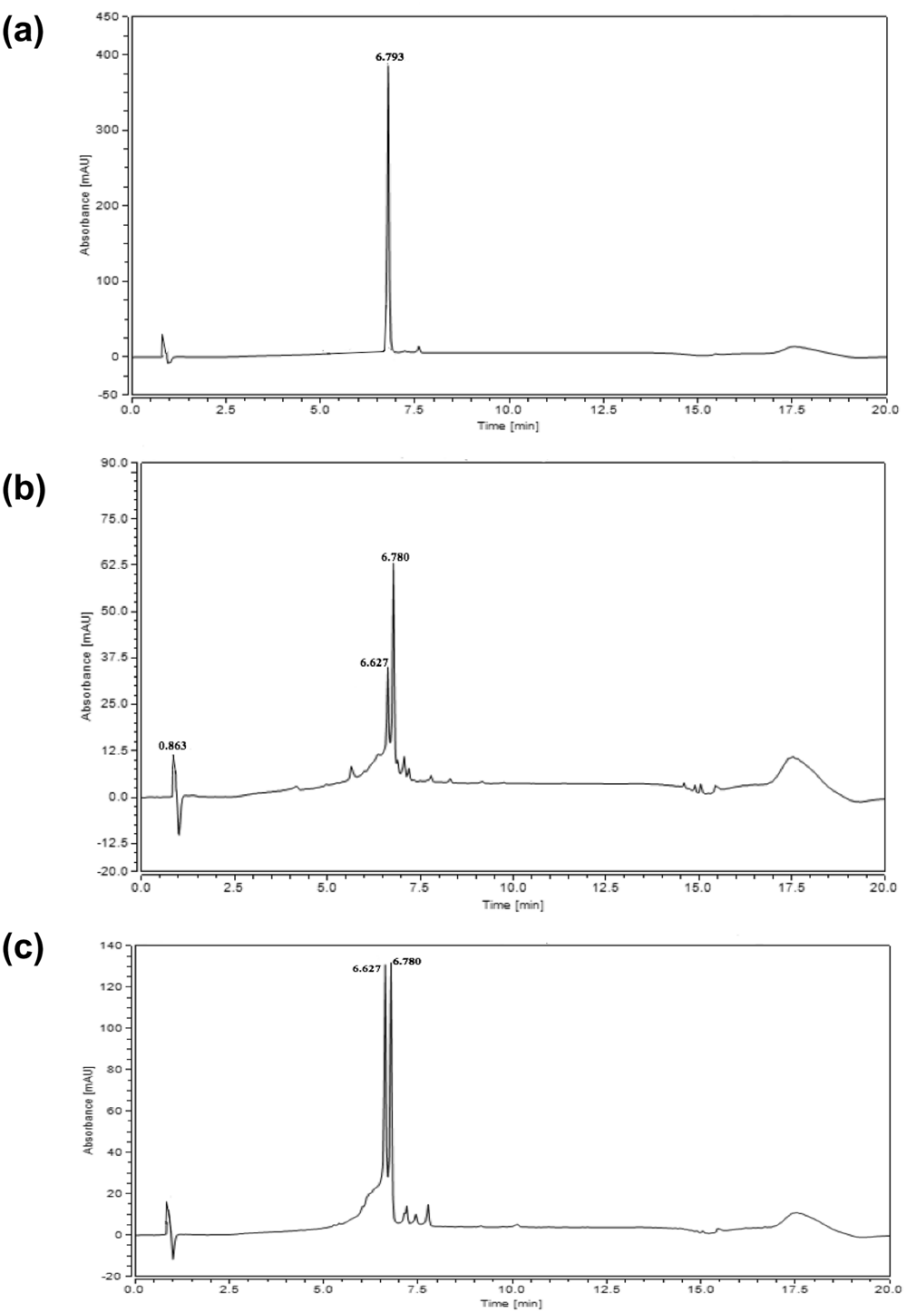
Chromatograms of 0.1 mg/ml Verbascoside (**a**) 1 mg/ml of *P. afra* L. extract (**b**) and 1 mg/ml *P. pysillium* L. extract (**c**); under optimum conditions using UPLC-DAD.

After selecting the optimum solvent, further investigation regarding the gradient time took place, till the optimum was reached, as illustrated in the experimental part. The temperature was adjusted at 35 °C, higher temperature didn’t improve the resolution, neither had a dramatic effect on the tR decrease. The wavelength was adjusted at 332 nm, as per the UV spectrum of VER determined by the diode array detector (DAD). VER standard was injected under the optimum conditions and its peak appeared at 6.793 min as in Fig. 4 (a).

The method was validated according to the ICH guidelines. To evaluate the linearity of the method, a calibration curve was constructed as mentioned in the experimental part. The method was linear over the concentration range of 6–400 μg/ml, the regression equation was y = 341.34 × −0.6013. The good linearity of the method was confirmed by the value of the regression coefficient (r) 0.9999. The limit of quantification was 4 μg/ml and the limit of detection was 1.35 μg/ml.

The repeatability of the method was evaluated by injecting the three concentrations (10, 20, and 40 μg/ml), three times each on the same day and the % RSD were 0.72, 1.63, and 0.33%, respectively. For the intermediate precision, the same three concentrations were injected each three times for three consecutive days and the % RSD were 1.54, 1.80, and 1.73%, respectively. The low values of the % RSD for both repeatability and intermediate precision indicate high precision of the method.

The accuracy of the method was determined by comparing the actual concentration found by the system to the theoretical value of ten standards of the calibration curve. And the % recovery ranged from 97.72 to 101.73%, with % RSD ranging from 0.09 to 1.91%.

The newly developed and validated method was applied on the identification and quantification of VER on *P. afra* L. and *P. pysillium* L. Accordingly, each plant extract was injected separately under the optimum conditions; the peak that appeared at tR 6.780 min corresponds to VER. This was identified by peak matching with VER standard, as presented in Fig. 4.

The constructed calibration curve was employed to quantify VER in the plant extracts. Hence, it was found that *P. afra* L. contains 19.01 μg VER/mg of extract, while *P. pysillium* L. contains 25.56 μg VER/mg of extract.

Additionally, both *P. pysillium* L. and *P. afra*, L. were spiked with VER standards at 3 different levels to further ensure the accuracy of the method and the quantification of VER.

As 1 mg *of P. afra* was found to contain 19.01 μg VER, accordingly, it was spiked with 10, 20, and 30 μg/ml, so that the final concentration is 29.01, 39.01, and 49.01 μg, respectively. The % recoveries were 96.28%, 97.13%, and 95.54% with % RSD 0.972%, 0.814%, and 1.12%, respectively. On the other hand, one mg *of P. psyllium* L. was found to contain 25.56 μg VER. Hence, it was spiked with 10, 20, and 30 μg/ml, so that the final concentration is 35.56, 45.56, and 55.56 μg, respectively. The % recoveries were 99.32%, 99.06%, and 95.48% with % RSD 0.79%, 2.09%, and 1.36%, respectively.

### Cytotoxicity and cell cycle analysis

**Figure 5:**
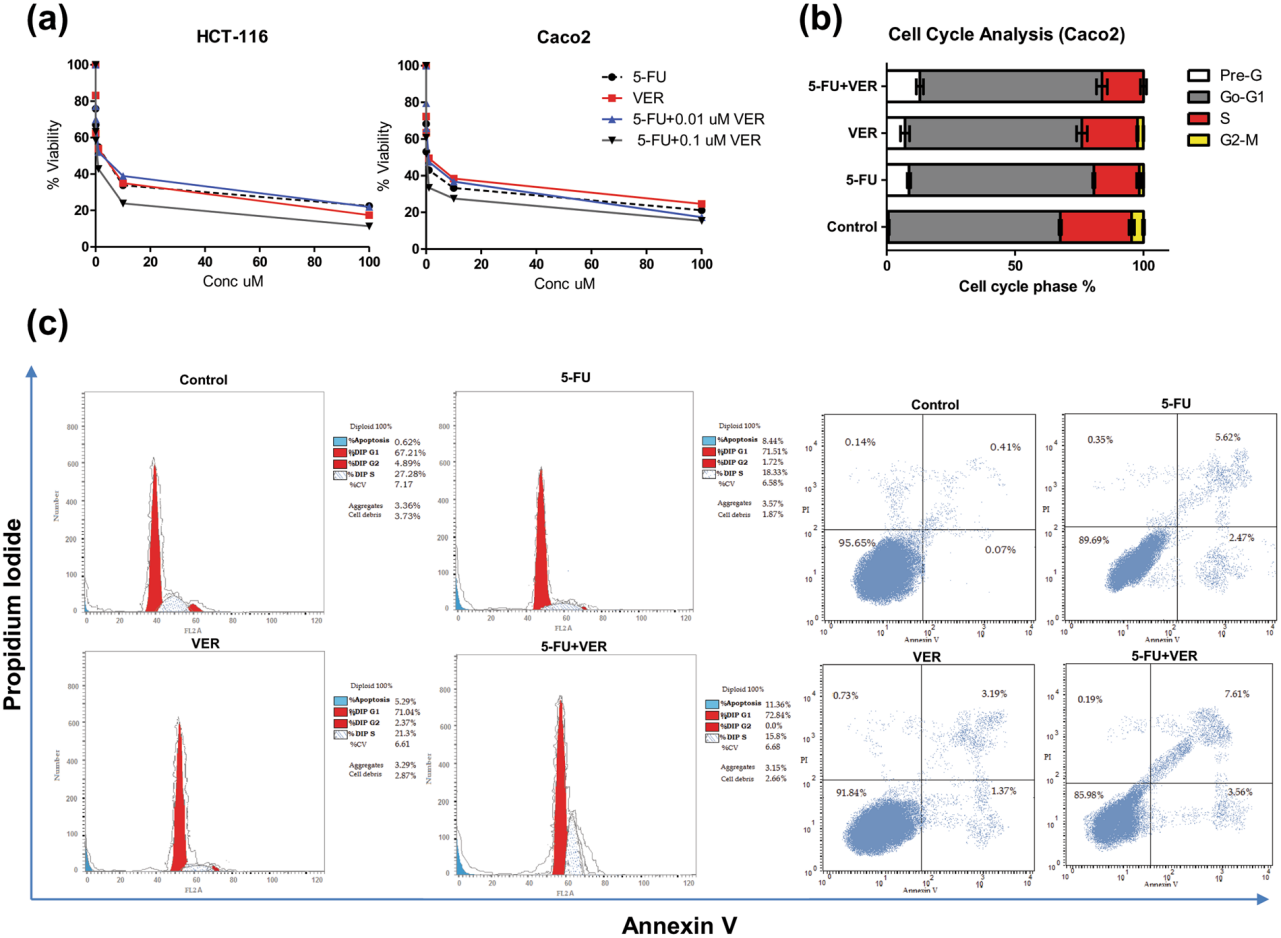
Effect of 5-FU and VER, alone and combined, on viability and cell cycle progression in Caco-2 & HCT-116 cell lines. (**a**) Dose-response plots of 5-FU, VER, and 5-FU + VER on HCT-116 and Caco-2 cell lines after 72 hrs exposure, as detected by MTT assay. (**b,c**) DNA content-based cell cycle analysis in Caco-2 cell line treated with 10 μM 5-FU, 0.1 μM VER, & 10 μM 5-FU + 0.1 μM VER. Results represent three independent experiments performed in triplicates.

MTT assay showed that 5-FU, VER, 5-FU + 0.01 µM VER, and 5-FU + 0.1 µM VER decreased the rate of cell proliferation in HCT-116 and Caco-2 cell lines, as compared to control (Fig. 5). The IC_50_ of 5-FU and VER was determined and found to be 1.199 and 1.088 µM on HCT-116 cells as well as 0.269 and 0.956 µM on Caco-2 cells, respectively. Combining a low dose of VER (0.01 μM) to 5-FU was found to produce an antagonistic interaction where the CI was found to be 3.45 and 1.25 on HCT-116 and Caco-2 cell lines, respectively. However, adding a high dose of VER (0.1 μM) to 5-FU had interestingly resulted in a synergistic interaction on both cell lines where the CI was found to be 0.43 and 0.25 on HCT-116 and Caco-2 cells, respectively (Table 2). Hence, treatment of Caco-2 cells with 5-FU+0.1 μM VER was used for further investigations. Moreover, as shown in Fig. 5, cell cycle analysis performed on Caco-2 cells exposed to 5-FU and VER, either alone or combined, caused cell cycle arrest at G1 with a 12.44-, 10.27-, and 18.49-fold increase in the cells in the pre-apoptotic phase after treatment with 10 μM 5-FU, 0.1 μM VER, and 10 μM 5-FU + 0.1 μM VER, respectively. In a previous study, VER was also found to significantly inhibit the proliferation of colorectal cancer cells^28^.

**Table 2:** The IC_50_ & combination index (CI) values for 5-FU, verbascoside, each alone & combined.

Drug/Combination	IC50 (µM)	CI	IC50 (µM)	CI
	HCT-116		Caco-2	
5-FU	1.199	—	0.2691	—
VER	1.088	—	0.9560	—
5-FU + 0.01 µM VER	1.482	1.25	0.9254	3.45
5-FU + 0.1 µM VER	0.1875	0.43	0.08619	0.25

### Effect on caspase-3, caspase-8, and caspase-9

**Figure 6:**
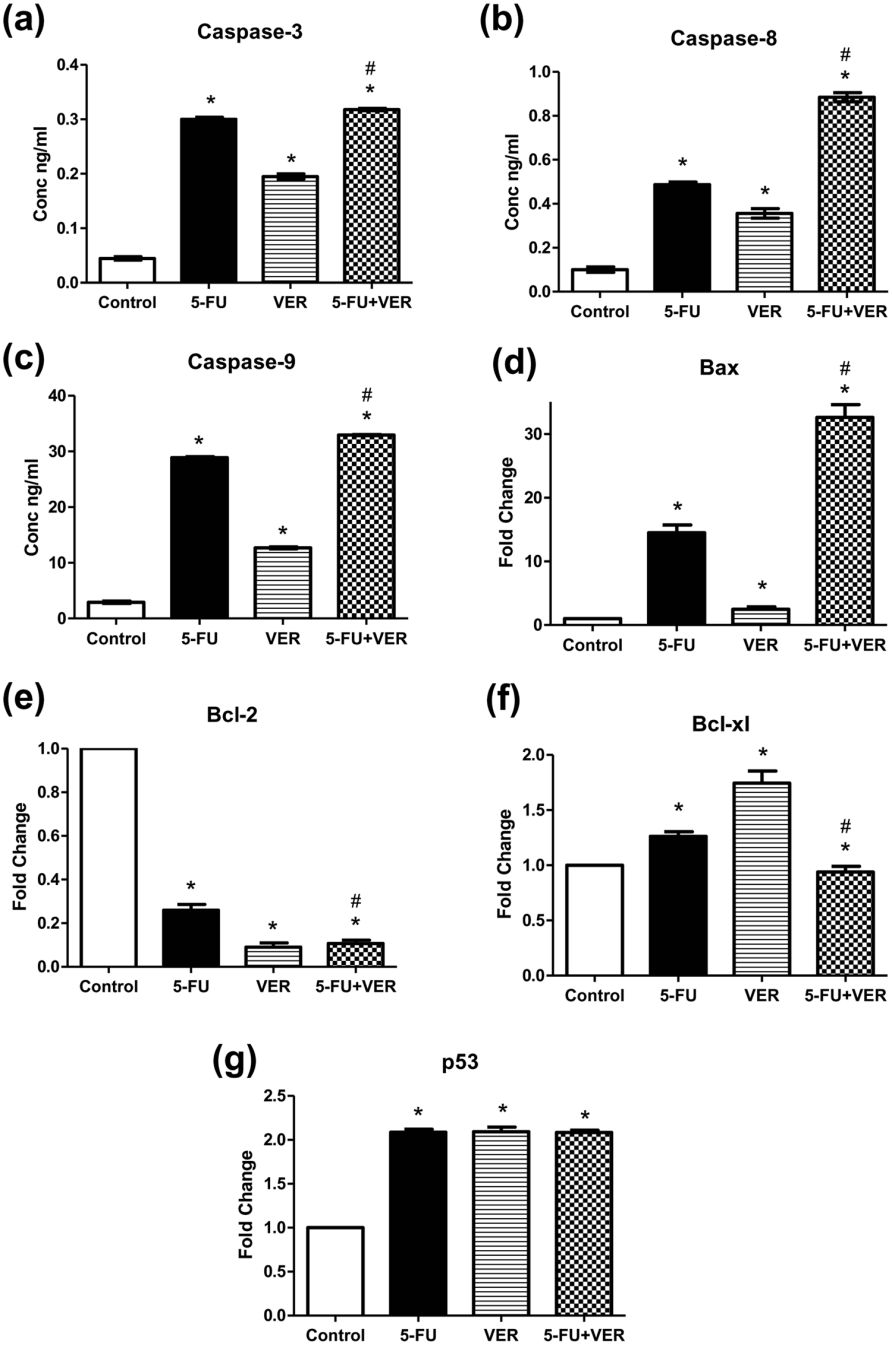
Effect of 5-FU and VER, alone and combined, on (**a**) caspase-3, (**b**) caspase-8, and (**c**) caspase-9 protein levels as well as (**d**) Bax, (**e**) Bcl-2, (**f**) Bcl-xL, & (**g**) p53 gene expression. Protein levels were estimated using ELISA. Gene expression levels were estimated using relative qRT-PCR method (fold change from control untreated samples normalized to GAPDH). Values are presented as means ± S.D. from three independent experiments performed in triplicates. *P < 0.05 significant from control untreated cells, ^#^P < 0.05 significant from 5-FU-treated cells using one way ANOVA followed by Bonferroni *post hoc* test.

As shown in Fig. 6, Treatment of Caco-2 with 5-FU, VER, and 5-FU + VER caused 6.7-, 4.3-, and 7.1-fold increase in the levels of caspase-3, a crucial mediator of apoptosis, as compared to control, respectively. This shows that combining VER to 5-FU caused a 1.1-fold further increase in caspase-3 levels compared to 5-FU alone. Moreover, in order to explore the mechanism of action by which apoptosis was initiated in cancer cells, the effect on caspase-8 and -9 was further investigated. Regarding caspase-8, treatment with 5-FU, VER, and 5-FU + VER caused a 4.8-, 3.5-, and 8.8-fold increase in its level as compared to control, respectively. Accordingly, the increase in caspase-8 in the 5-FU + VER-treated group was 1.8-fold higher compared to 5-FU alone. As for caspase-9, 5-FU, VER, and 5-FU + VER showed a 9.9-, 4.4-, and 11.3-fold higher expression levels compared to control, respectively, with a 1.8-fold higher expression reported in the combination group compared to 5-FU-treated group.

In human cells, induction of apoptosis involves either an intrinsic or extrinsic pathway. These pathways are triggered and induced either through Fas (extrinsic) or Bax (intrinsic). Fas activates pro‐caspase‐8^29,30^ which then activates caspase‐8 to cleave and further activate caspase-3 and other downstream caspase enzymes. Cytochrome-c is then released by the mitochondria under the influence of the pro-apoptotic Bax^31^. Pro‐caspase‐9, the initiator of the intrinsic apoptosis pathway, then becomes activated by cytochrome-c, consequently activating caspase‐3 beside other caspases^32,33^. Hence, the results of the present study might suggest the involvement of both intrinsic and extrinsic pathways. Further insights into the apoptotic mechanism of action will be discussed later.

### Effect on Bax, Bcl-2, BclxL, and p53 gene expression

For further assessment of the apoptotic potential of the combination treatment, the gene expression of Bax, Bcl-2, Bcl-xL, and p53 was estimated in Caco-2 cells treated with 5-FU, VER, alone and combined (Fig. 6). Regarding Bax gene expression, the present results showed that treatment with 5-FU, VER, and 5-FU + VER caused a 14.48-, 2.46- and 32.59-fold higher expression than control untreated cells, respectively, where the combination-treated cells expression was 2.25-fold higher than that in 5-FU-treated cells. Our results also showed that Bcl-2 expression was decreased in 5-FU-, VER-, and 5-FU + VER-treated cells by 74, 91, and 89.33%, as compared to control untreated cells, respectively. Additionally, the combination treatment showed a 58.96% decrease in Bcl-2 expression, as compared to 5-FU alone. On the other hand, the expression of Bcl-xL showed no significant difference in the combination treated group compared to control in spite the fact that both 5-FU and VER alone showed 1.26- and 1.74-fold increase in the expression compared to control, respectively. Moreover, the levels of p53 was almost 2-fold higher in all treated groups (with no significant differences between them) compared to control. These results are in accordance with the study of Zhou *et al*.^28^ and strongly suggest a potential apoptotic effect of VER in colorectal cells especially when combined with 5-FU. Thus, it can be concluded that the apoptotic potential of VER, especially when combined with 5-FU, could be primarily due to its effect on Bax and Bcl-2 rather than Bcl-xL and p53.

### Effect on PI3k inhibition

**Figure 7:**
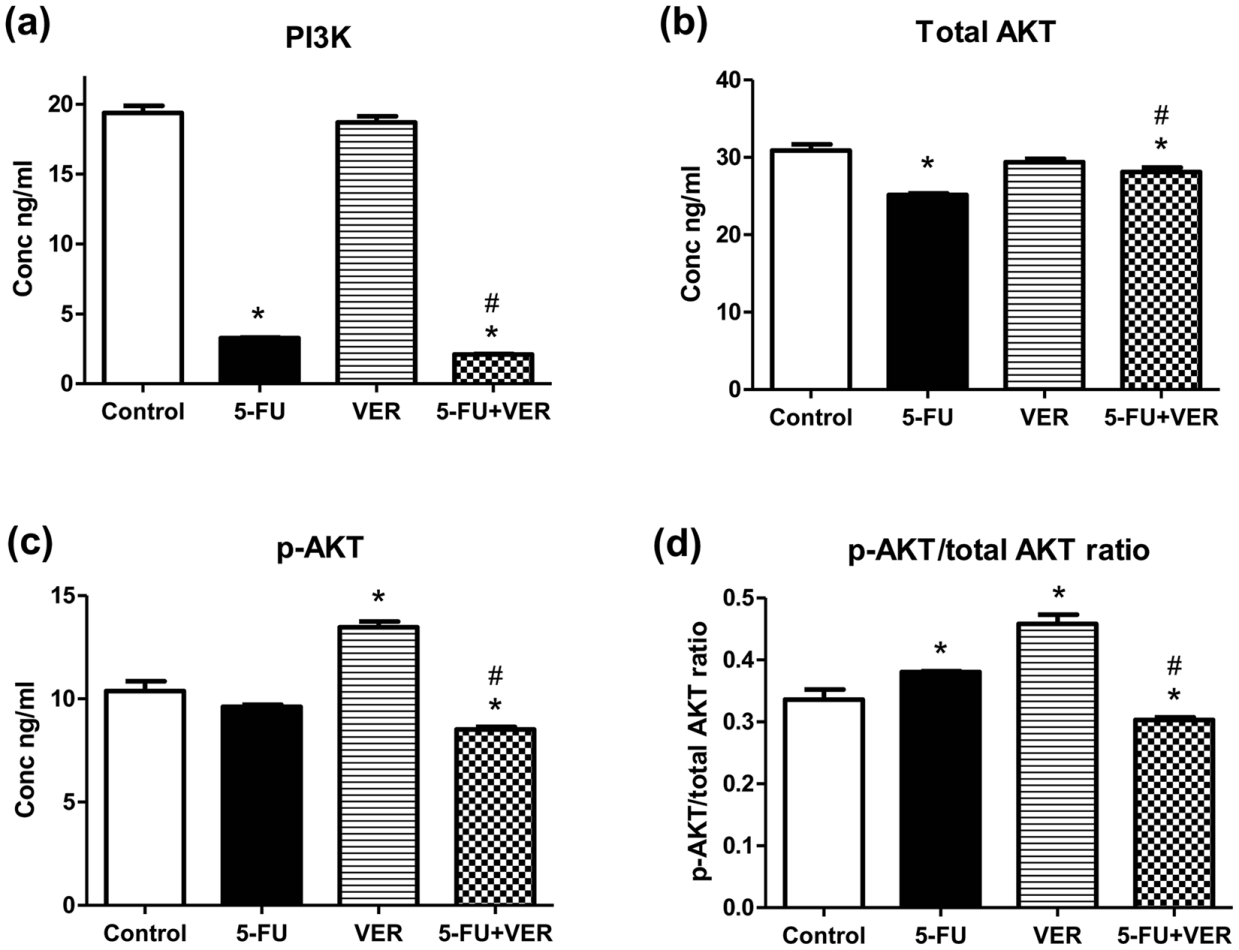
Effect of 5-FU and VER, alone and combined, on (**a**) PI3K, (**b**) total AKT, (**c**) p-AKT, & (**d**) p-AKT/total AKT ratio. PI3K, total AKT, & p-AKT were determined using ELISA. Values are presented as means ± S.D. from three independent experiments performed in triplicates. *P < 0.05 significant from control untreated cells, ^#^P < 0.05 significant from 5-FU-treated cells using one way ANOVA followed by Bonferroni *post hoc* test.

As shown in Fig. 7, VER showed no change in PI3K level compared to control, however, cells treated with 5-FU caused a 83.18% reduction in PI3K compared to untreated cells. Moreover, combining VER to 5-FU caused a further significant reduction in PI3K reaching 89.14 and 35.59%, as compared to control and 5-FU, respectively.

There is no evidence in the literature for the effect of VER on PI3K/AKT pathway except for the study of Wang *et al*.^34^, which reported an upregulation of PI3K by VER in neurons suggesting a neuroprotective role against beta-amyloid-induced neurotoxicity, however, no reports for an influence on tumor cells, in general, or CRC cells, in particular were found. To our knowledge, the present study offers the first evidence for a potential effect of VER, particularly when combined with 5-FU, on PI3K/AKT pathway in CRC. Although, VER alone failed to inhibit PI3K contrary to 5-FU, it unexpectedly caused a significant downregulation in its level when combined to 5-FU. These findings need further studies to explore the exact mechanism by which VER potentiated the effect of 5-FU on PI3K when used in combination.

### Effect on total AKT, p-AKT, and p-AKT/total AKT ratio

Total AKT was reduced in the cells treated with 5-FU and 5-FU + VER, however, the VER-treated cells showed higher AKT expression compared to control. Interestingly, different pattern was observed for p-AKT where the expression in 5-FU-treated cells was the same as that of control while VER-treated cells showed higher expression than that in untreated control cells. The combination, however, showed the lowest levels of p-AKT as compared to control, 5-FU-, and VER-treated cells. In order to provide a more clear picture of the observed results, the p-AKT/total AKT ratio was calculated where 5-FU- and VER-treated cells showed 1.13- and 1.36-fold higher ratio compared to control, however, the combination treatment showed more than 1 fold lower ratio than that of the control untreated cells (Fig. 7). Although, 5-FU remains a common treatment for patients with colon cancer, resistance to the drug was widely reported leading to therapeutic failure^35^. Resistance of CRC cells was previously linked to the upregulation of PI3K/AKT pathway in these cells and hence, suppressing this pathway has been suggested for sensitizing cancer cells to conventional treatment^36,37^. These findings are in agreement with our results where 5-FU caused the p-AKT/total AKT ratio to increase significantly compared to control implying resistance of CRC to 5-FU. In spite the fact that VER, when used alone, showed a similar pattern to that of 5-FU, the combination showed a different pattern where the p-AKT/total AKT ratio was significantly decreased compared to untreated cells. As mentioned earlier regarding the pattern observed with PI3K, further studies are recommended in order to unravel the mechanism by which VER synergized with 5-FU to decrease the p-AKT/total AKT ratio and hence cause an overall downregulation of the PI3K/AKT pathway leading to sensitization of CRC cells to 5-FU.

In conclusion, the present findings suggest a potential role of VER in reducing the resistance of CRC to 5-FU *via* targeting the PI3K/AKT signaling pathway. Further studies, however, are recommended to validate the *in vivo* efficacy of the combination.

## Data Availability

The datasets generated during and/or analysed during the current study are available from the corresponding author on reasonable request.
