# Author Correction: Verbascoside: Identification, Quantification, and Potential Sensitization of Colorectal Cancer Cells to 5-FU by Targeting PI3K/AKT Pathway

**DOI:** 10.1038/s41598-019-55121-x

**Published:** 2019-12-04

**Authors:** Yasmeen M. Attia, Dina M. El-Kersh, Hebatallah A. Wagdy, Mohamed M. Elmazar

**Affiliations:** 1 Pharmacology Department, Faculty of Pharmacy, The British University in Egypt, El-Sherouk City, P.O. Box 43, Cairo, 11837 Egypt; 2 Pharmacognosy Department, Faculty of Pharmacy, The British University in Egypt, El- Sherouk City, P.O. Box 43, Cairo, 11837 Egypt; 3 Pharmaceutical Chemistry Department, Faculty of Pharmacy, The British University in Egypt, El-Sherouk City, P.O. Box 43, Cairo, 11837 Egypt; 4 The Center for Drug Research and Development (CDRD), Faculty of Pharmacy, The British University in Egypt, El-Sherouk City, P.O. Box 43, Cairo, 11837 Egypt

Correction to: *Scientific Reports* 10.1038/s41598-018-35083-2, published online 16 November 2018

This Article contains a typographical error in the Results and Discussion section under subheading ‘Identification and suggested fragmentation of verbascoside’ where,

“The presence of molecular ion at m/z 623 (C_29_H_35_O_15_)^−^ for VER [M-H]^−^ structure whereas characteristic ion peak of the phenyl propanoid moiety recognized at m/z 161 [caffeic acid-H-H_2_O]^−^ was due to loss of H_2_O from caffeic acid at m/z 179 [C_9_H_7_O_4_]^−^ and the ion peak at m/z 461 represents [M-H-hexose sugar]^−^ due to loss of a hexose sugar (−162 amu)^25–27^”

should read:

“The presence of molecular ion at m/z 623 (C_29_H_35_O_15_)^−^ for VER [M-H]^−^ structure whereas characteristic ion peak of the phenyl propanoid moiety recognized at m/z 161 [caffeic acid-H-H_2_O]^−^ was due to loss of H_2_O from caffeic acid at m/z 179 [C_9_H_7_O_4_]^−^ and the ion peak at m/z 461 represents [M-H-Caffeoyl moiety]^−^ due to loss of a Caffeoyl moiety (−162 amu)^25–27^”

Additionally, in Figure 3, the label on the peak is incorrectly labelled as “[M-H-caffaeoyl-rhamnose]”. The correct Figure 3 appears below as Figure 1.

**Figure 1:**
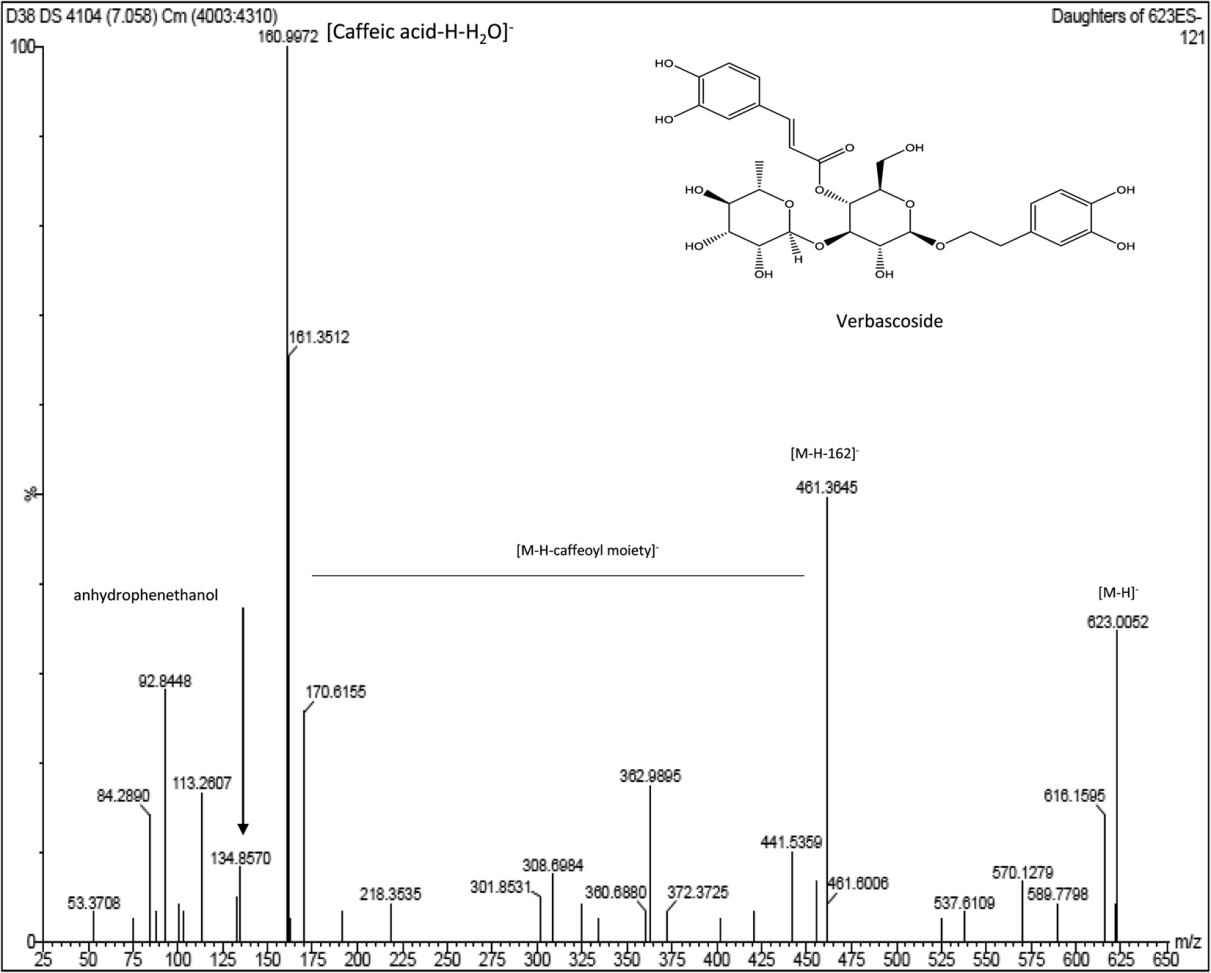
.

